# Nutritional and Phytochemical Traits of Apricots (*Prunus Armeniaca* L.) for Application in Nutraceutical and Health Industry

**DOI:** 10.3390/foods10061344

**Published:** 2021-06-10

**Authors:** Omar Alajil, Vidya R. Sagar, Charanjit Kaur, Shalini Gaur Rudra, R. R. Sharma, Rajeev Kaushik, Mahendra K. Verma, Maharishi Tomar, Manoj Kumar, Mohamed Mekhemar

**Affiliations:** 1 Division of Food Science & Postharvest Technology, ICAR–Indian Agricultural Research Institute, New Delhi 110012, India; omar8alajil@gmail.com (O.A.); sagarvrpht@gmail.com (V.R.S.); charanjitkaur6@gmail.com (C.K.); gaurshalini@gmail.com (S.G.R.); rrs_fht@rediffmail.com (R.R.S.); 2 Division of Microbiology, ICAR–Indian Agricultural Research Institute, New Delhi 110012, India; rajeev_micro@iari.res.in; 3 Division of Fruits & Horticultural Technology, ICAR–Indian Agricultural Research Institute, New Delhi 110012, India; mkverma@iari.res.in; 4 ICAR–Indian Grassland and Fodder Research Institute, Jhansi 284003, India; maharishi89@gmail.com; 5 Chemical and Biochemical Processing Division, ICAR–Central Institute for Research on Cotton Technology, Mumbai 400019, India; 6 Clinic for Conservative Dentistry and Periodontology, School of Dental Medicine, Christian-Albrecht’s University, 24105 Kiel, Germany

**Keywords:** apricots, antioxidant activity, sugars, organic acids, minerals, phytochemicals

## Abstract

Apricot (*Prunus armeniaca* L.) is a nutritious fruit, rich in bioactive compounds, known for their health benefits. The present study attempts to evaluate nutritional (sugars, organic acids, minerals) and nutraceutical traits (total phenolics, flavonoids, carotenoids, antioxidant activity) of six commercial apricot genotypes grown in India. Antioxidant activity was determined using three in-vitro assays, namely CUPRAC (cupric reducing antioxidant capacity), FRAP (ferric reducing antioxidant power) and DPPH (1,1-diphenyl-2-picryl-hydrazyl). Significant (*p* < 0.05) differences were observed in the genotypes concerning nutritional and nutraceutical traits. Sucrose accounted for more than 60% of total sugars in most genotypes, followed by glucose and fructose. Citric acid accounted for more than 50% of the total organic acids present, followed by malic and succinic acids. Apricot is a good source of potassium (1430.07 to 2202.69 mg/100 g dwb) and iron (2.69 to 6.97 mg/100 g dwb) owing to its mineral composition. Total carotenoids content ranged from 0.44 to 3.55 mg/100 g, with β-carotene accounting for 33–84% of the total content. The results strongly suggest that genotypes ‘CITH-A-1’ and ‘CITH-A-2’, which have high dry matter and carotenoids content, are well suited for drying. ‘Roxana’ and ‘CITH-A-3’ are great for fresh consumption, while ‘Shakarpara’ and ‘Gold Cot’ are excellent for juice processing.

## 1. Introduction

Apricot enjoys an eminent position amongst the common temperate fruit grown across the globe and is the 3rd most economically important stone fruit after plum and peach [1]. The total global apricot production is 3.8 million tonnes, with Turkey (730,000 tonnes) being the largest producer, followed by Uzbekistan (490,000 tonnes) and Iran (340,000 tonnes) [2]. Apricot production in India has nearly doubled in the last 20 years, reaching 15 thousand tonnes per year [2].

Apricot is coined as the ‘golden fruit’ from the viewpoint of its nutritional value and medicinal properties. The fruit contains a high concentration of bioactive phytochemicals such as carotenoids, flavonoids, phenolics, and antioxidants and is regarded as a functional food [3]. The fruit has a distinct flavour, a heavy fragrance, and an appealing yellow to orange colour with a reddish random overlay [4]. The fruit is typically consumed fresh, but it can also be processed into jam, juice, and dried fruits by sun drying [5].

The major determinants of taste, colour, and nutrition in apricots are phytochemicals, and their content is directly impacted by genotype, ripening stage environmental conditions, and cultivation practises [1]. Apricots are high in phenolics; the main phenolics are chlorogenic, gallic, ferulic, caffeic, 4-aminobenzoic, procatechin, salicylic, and *p*-coumaric acid, and the major flavonols are quercetin, glycoside rutin, resveratrol, and vanillin [6].

These fruit are also rich in carotenoids, including β-carotene, γ-carotene, lycopene, β-cryptoxanthin, phytoene, phytofluene, and lutein [7,8,9]. β-carotene has potent antioxidant activity and is proven to provide important health benefits such as reducing oxidative stress, boosting the immune system, decreasing the risk of heart disease and some forms of cancer, and protecting against age-related macular degeneration [8,9].

Sugars and organic acids are essential primary metabolites in apricots that are linked with nutrition and the fruit’s delicate fragrance [9]. Sucrose is the most abundant sugar, followed by glucose, fructose, and sorbitol [10]. Sucrose and fructose are important components of fruit sweetness, fragrance, and customer satisfaction. Citric and malic acids predominate in apricot, with minor quantities of quinic, succinic, and ascorbic acids [11].

Due to favourable eco-geographical and climatic conditions, the northern Indian region (i.e., hills of Himachal Pradesh, Jammu and Kashmir, and Uttar Pradesh) is well suited for apricot production. The area has a rich diversity, characterized by self-incompatibility, small to medium-sized fruits, a long ripening time, and high chilling requirements [1]. A plethora of publications report the chemical composition of apricots from various regions of the world [7,12,13,14,15], but there are few reports on the nutritional diversity of apricots from India [1,10,16], and a thorough study is still warranted. As a result, the current study was planned to evaluate the nutritional (sugars, organic acids, minerals) and nutraceutical properties of new Indian genotypes with those of European genotypes grown in north India. Breeders, horticulturists, and processing units can benefit from information on the quality and processing characteristics of apricot genotypes in order to identify potential genotypes suitable for the fresh consumer market as well as the processing industry.

## 2. Material and Methods

### 2.1. Experimental Material

**Figure 1:**
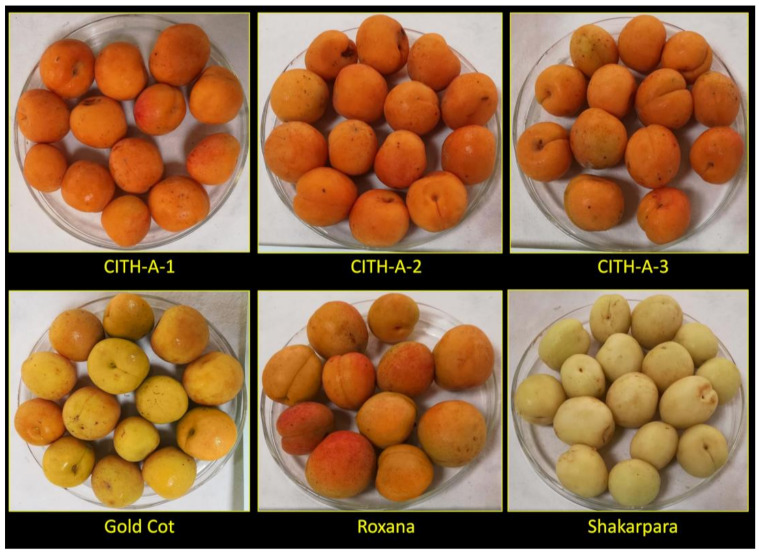
Variation in skin colour of different apricot genotypes.

Six apricot (*Prunus armeniaca* L.) genotypes, including three Indian genotypes (*viz*., ‘CITH-A-1’, ‘CITH-A-2’, and ‘CITH-A-3’) and three European genotypes (viz., ’Roxana’, ‘Gold Cot’, and ‘Shakarpara’), were provided by the Regional Horticultural Research Station, Bajaura, Himachal Pradesh, India on June 2019 (Figure 1). The research field was located at 31°10′ N, 77°6′ E and 1090 m above mean sea-level. The harvesting of the apricot fruit was performed in the same period with the same degree of maturity. The sample was representative of a total of 120 fruit which were collected from six trees in the research field and then sorted and cleaned. Mature, healthy fruits were transported to the Division of Food Science and Postharvest Technology, ICAR–Indian Agricultural Research Institute, New Delhi, in a refrigerated van for analysis. The fruit were washed, air-dried, and deseeded. Later, pulp along with peel was crushed and converted into fine pulp and stored at −80 °C until the nutritional and nutraceutical content was determined. Whole fruit along with peels was used to prepare a homogenous sample; since, peels are usually consumed with the apricot fruit.

### 2.2. Estimation of Quality Traits

Dry matter was determined by placing 5 g of pulp in a hot air oven at 60 °C till constant weight was achieved. The TSS (total soluble solids) was measured using a digital hand refractometer (ATAGO CO. LTD., Tokyo, Japan) and expressed as °Brix. The TA (titratable acidity) of fruit pulp was determined by titrating the pulp extract against sodium hydroxide and expressed as a percentage of malic acid [17].

### 2.3. Estimation of Colour

A colour meter was used to determine the colour of the fruits (ColorFlex EZ, Hunter Associates Laboratory, Inc., Mumbai, India). The results were expressed in terms of CIE L*, a*, b*, hue angle (h°), and chroma. The values of hue angle (h° = arctan (b*/a*)) and chroma (C* = (a*^2^ + b*^2^)^0.5^) were computed from a* and b* values.

### 2.4. HPLC Estimation of Sugars and Organic Acids

Standards of sugars (sucrose, glucose, and fructose) and organic acids (citric acid, malic acid, succinic acid, and ascorbic acid) were purchased from Sigma. The HPLC system (Waters India Pvt Ltd., New Delhi, India) comprised of a binary pump (model 515), refractive index (2414), photodiode array detector (PDA, 2998), and an Aminex HPX-87H column (Bio-Rad Laboratories, Hercules, CA, USA). The separation was carried out in isocratic mode with 5 mM H_2_SO_4_ as mobile phase flowing at 0.5 mL/min and a column temperature of 50 °C. For the identification of individual organic acids and sugars, PDA (210 nm) and RI detectors were used in sequence. Individual sugars and organic acids were quantified by comparing their corresponding peaks to those of a standard as a function of retention time and peak area.

The fruit sweetness index (SI) was calculated according to Roussos et al. [15], the relative quantities of individual sugars and their sweetness properties were computed as follows:

Sweetness index (SI) = 1 × glucose conc. + 1.35 × sucrose conc. + 2.30 × fructose conc.

### 2.5. Estimation of Total Carotenoids

Total carotenoids content of apricot genotypes was determined using the method reported by Rodriguez-Amaya and Kimura [18] with slight modification. Five grammes of the sample were extracted with 15 mL of acetone, and the extract was transferred to a 500 mL separation funnel. This process was repeated three times, or until the sample residues became colourless. After that, 40 mL of petroleum ether was added. The acetone aqueous phase was eliminated by slowly adding milli-Q water, separating the mixture into two layers, and discarding the bottom layer. This process was repeated three times until no residual acetone remained. The extract was then passed through 15 g of anhydrous sodium sulphate to remove the aqueous phase completely. Petroleum ether was used to make up the volume, and the samples were read at 450 nm. Results were expressed in terms of mg per 100 g.

### 2.6. HPLC Estimation of β-Carotene

The petroleum ether fraction from the previous carotenoids extract was evaporated to dryness in a rotary vacuum evaporator at 40 °C. The residue was reconstituted with 2 mL of methyl tert-butyl ether (MTBE) and filtered into an HPLC glass vial using a 0.22 m syringe filter. The HPLC measurement of carotenoids was performed as per method described by Huang et al. [19] with small modifications, using a UHPLC system (Dionex UltiMate 3000, Thermo Fisher Scientific, Bremen, Germany) fitted with a YMC C30 RP column (250 × 4.6 mm, 5 µm; Waterco., Milford, MA, USA) held at 20 °C, 500G pump, and S-3210 PDA detector. The chromatography was performed in gradient mode with two buffers, buffer A (methanol containing 1% (*v*/*v*) double-distilled water and 0.01% (*w*/*v*) ammonium acetate) and buffer B (100% MTBE), and a linear gradient from 0 to 90% B over 40 min at a flow rate of 1 mL/min, with detection at 450 nm. The quantification of β-carotene was accomplished by comparing the corresponding peak to that of the standard as a function of retention time and peak area. The parameters are presented in mg of β-carotene per 100 g.

### 2.7. Determination of Proximate Content and Minerals

The AOAC methods were used to determine the proximate composition of apricot genotypes. The protein content was estimated using the Kjeldahl distillation apparatus, the total nitrogen was estimated and then converted to protein using the conversion factor (N × 6.25). Crude fat was determinated by solvent extraction in a Soxhlet apparatus. The crude fibre content was determined after sample digestion with 2.5 M H_2_SO_4_ and 2.5 M NaOH, respectively. The samples were then filtrated and dried in a hot air oven at 100 °C, before being incinerated at 600 °C in a muffle furnace till constant weight was obtained. The ash content was estimated by incineration of samples in a muffle furnace at 550 °C for 5–6 h until constant mass was achieved.

### 2.8. Determination of Mineral Composition

The ash obtained in previous step was dissolved in 50 mL of 5% HCl, and the volume was used to determine mineral elements (Fe, Zn, Mn, and Cu) using atomic absorption spectroscopy (AAS, AA4000, Spectrum-SP, Darmstadt, Germany), sodium (Na) and potassium (K) using a flame photometer (128, Systronics, Ahmedabad, India), and phosphorus (P) using a UV–VIS spectrophotometer [20]. The results were given in mg per 100 g dried weight basis (dwb).

### 2.9. Estimation of Total Phenolics, Flavonoids, and Antioxidants Activity

Two grams of pulp sample were extracted with 10 mL of 80% methanol, sonicated for 30 min, then centrifuged for 10 min at 10,000× *g* and 4 °C, the supernatant was collected, and the residue was extracted twice in the same manner. All extracts were combined, and the volume was reduced to 30 mL before being filtered through a nylon filter (0.22 m) for estimation of total phenolics, total flavonoids, and antioxidant assays.

Total phenolics content (TPC) was estimated according to Singleton et al. [21], using the Folin–Ciocalteu reagent (FCR). In total, 100 µL of the previous extract was mixed with 2.9 mL of deionized water, 0.5 mL of FCR, and 2 mL of Na_2_CO_3_ (20%). The mixture was incubated at room temperature for 60 min before being measured for absorbance at 750 nm with a UV–VIS spectrophotometer (Varian Cary 50). The results were expressed in milligrams of gallic acid equivalents (GAE) per 100 g.

The TFC (total flavonoids content) was determined using the method described by Zhishen et al. [22]. A known volume (0.3 mL) of sample extract was mixed with 0.3 mL of NaNO_2_ (5%) before adding 0.3 mL of AlCl_3_ (10%) and 2 mL NaOH (1 M). The mixture was thoroughly homogenised until a pink to yellow colour appeared. A UV–VIS spectrophotometer (Varian Cary 50) was used to measure absorbance at 510 nm. The results were expressed as mg of catechin equivalent (CE) per 100 g.

The antioxidant activity was estimated using three assays, namely CUPRAC, DPPH, and FRAP. The cupric reducing antioxidant power assay (CUPRAC) was determined according to Apak et al. [23]. The assay consisted of mixing 0.1 mL of sample extract and 1 mL each of CuCl_2_ solution (1.0 × 10^−2^ mol/L), neocuproine alcoholic solution (7.5 × 10^−3^ mol/L), and NH_4_Ac (1 mol/L, pH 7.0) buffer solution, and water to make the final volume 4.1 mL. The mixture was incubated for 30 min, and absorbance was recorded at 450 nm against the reagent blank. The results were expressed as μmol Trolox equivalent (TE) per g.

The DPPH (2,2-diphenyl-1-picrylhydrazyl) assay was used to measure free radical scavenging activity [24]. Briefly, a DPPH solution of 0.0634 mmol/L was prepared in 70% methanol (*v*/*v*). An amount of 3.9 mL of DPPH solution was mixed with 0.1 mL of sample extract and vigorously shaken. The mixture was incubated in the dark for 30 min before measuring the difference in absorbance of the sample extract versus the control at 517 nm using a UV–VIS spectrophotometer. The results were expressed in terms of μmol TE per g.

The ferric reducing antioxidant power (FRAP) assay was estimated by the method described by Benzie and Strain [25]. A known amount of sample extract (0.1 mL) was introduced to 3 mL of FRAP reagent. The mixture was vortexed and then incubated for 4 min in a 37 °C water bath. The absorbance was measured at 593 nm using a UV–VIS spectrophotometer (Varian Cary 50). The findings were expressed as μmol TE per g.

### 2.10. Heat Map Analysis

The heat map depicting the relative content of each attribute in each variety and illustrating the Pearson’s correlation coefficient between selected attributes was generated using the MetaboAnalyst 3.0’s statistical package [26].

### 2.11. Statistical Analysis

All analyses were performed in triplicate, and the results were presented as mean values standard deviation. With the help of the SPSS (20.0) software, the analysis of variance (ANOVA) was performed to identify significant differences (*p* < 0.05) among the apricot genotypes, and the post hoc Duncan test was performed for a pair-wise comparison of genotypes for each of the parameters. Pearson’s correlation coefficient was used to show the relationship between selected traits. The correlation analysis was carried out with jamovi version 1.2.27 and a 5% degree of significance.

## 3. Results and Discussion

### 3.1. Fruit and Stone Weight

The physicochemical composition of apricot genotypes is presented in Table 1. Fruit weight is an important economic characteristic, and large-sized fruit attract a high market price. The fruit and stone weight ranged from 20.13 to 38.18 g and 1.62 to 2.96 g, respectively, according to our results. The genotype ‘Gold Cot’ had the most fruit and stone weight, while ‘Shakarpara’ had the least. Gupta et al. [27] previously recorded fruit and stone weight variations in the range from 8.0 to 15.1 g and 1.78 to 1.92 g, respectively, which are lower than our values. However, apricot genotypes grown in China had a higher fruit weight value (51.1 to 119.9 g). Similarly, many researchers have previously identified variations in apricot fruit weight [5,11,13].

### 3.2. Dry Matter

Apricot dry matter (DM) content is a significant factor that determines the fruit’s susceptibility to handling and transportation, as well as its suitability for processing and dehydration [28]. The DM content of apricots ranged significantly (*p* < 0.05) from 13.07% in ‘Shakarpara’ to 19.11% in ‘CITH-A-2’ (Table 1). In general, genotypes with low DM are preferred for fresh consumption [28], whereas those with high DM are best suited for drying and processing. Our findings are consistent with those published for apricot genotypes grown in Greece (9.6 to 15.9%) [13], Pakistan (14.7 to 21.2%) [14], and Turkey (11.8 to 25.8%) [28]. The observed differences may be attributed to genetics, climatic conditions, and cultivation practices [14].

### 3.3. Total Soluble Solids (TSS)

TSS ranged from 12.13 to 17.82 Brix, indicating significant genotype variation (Table 1). The highest content was found in ‘CITH-A-1’ (17.82° Brix), and the lowest in ‘Shakarpara’ (12.13° Brix). These results are consistent with previous findings for apricot genotypes grown in Greece, Shimla (India), and Italy, where TSS ranged from 11.5 to 19.3° Brix [12], 10.7 to 19.6° Brix [15], and 11.9 to 16.3° Brix [3,4], respectively.

### 3.4. Titratable Acidity (TA)

The titratable acidity (%) ranged from 1.88 in ‘CITH-A-2’ to 2.53 in ‘Gold Cot’ (Table 1). Our results are comparable to those published in Greece (0.27 to 1.91%) [12] and Spain (0.77 to 2.39 g/100 mL) [29]. The relationship between TSS and TA is an excellent predictor of fruit ripening and eating efficiency. TSS/TA values ranged from 5.18 in ‘Gold Cot’ to 9.47 in ‘CITH-A-2’. Fruit with a TSS/TA ratio of 10 to 15 are considered to be of acceptable eating quality [30].

### 3.5. Fruit Colour

Fruit colour is a significant predictor of ripening stage and carotenoids content [7,13], as well as a discriminating factor that determines customer acceptability. In the fresh market, consumers favour genotypes with appealing colours when making a purchasing decision. In contrast, colour is an essential element in industry for sorting, grading, and directing to subsequent process. In the current study, genotypes differed significantly (*p* < 0.05) in terms of colour attributes (Table 2). The values of L* (lightness) ranged from 52.10 to 71.51. ‘Shakarpara’ had the highest L* value among genotypes, while ‘CITH-A-1’ had the lowest. Similarly, the a* (redness) value ranged from 1.03 to 39.85. ‘CITH-A-2’ had the highest value among genotypes, while ‘Shakarpara’ had the lowest. The b* value (yellowness) ranged between 40.56 and 62.94. Genotype ‘Gold Cot’ had the highest b* value among genotypes, while ‘Shakarpara’ had the lowest. The determined colorimetric indices C* and h, which were derived from a* and b*, ranged from 40.59 to 69.48 and 54.84 to 88.43, respectively. Previous research in apricots revealed a strong inverse relationship between h◦ and fruit carotenoids content. Apricot genotypes with the lowest h◦ values may be defined as a carotenoids-rich source [7].

### 3.6. Soluble Sugars

**Figure 2:**
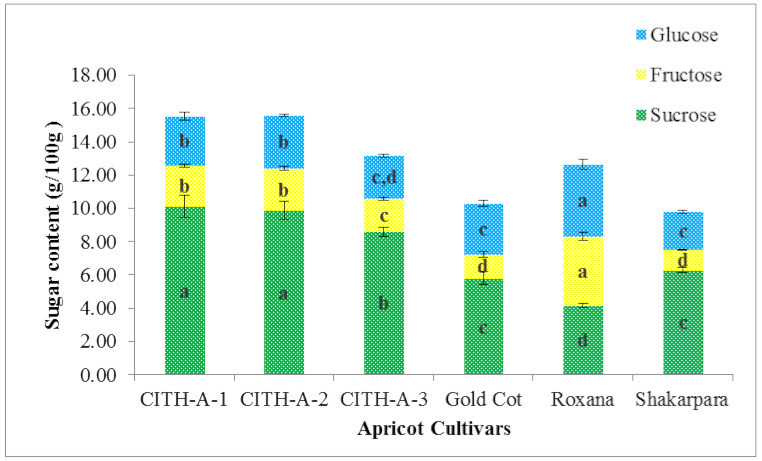
Sugar content of apricot genotypes from India. The same letter (a, b, c or d) inside a similarly coloured block indicates no statistically significant differences in values (*p* < 0.05).

Total sugar content ranged from 9.79 to 15.59 g/100 g, with significant differences (*p* <0.05) between genotypes (Table 3 and Figure 2). As shown in Figure 2, sucrose was the dominant sugar, accounting for more than 63% of total sugars and ranging from 4.15 to 10.13 g/100 g, glucose contributed about 22% of total sugars and ranged from 2.28 to 4.31, and fructose contributed about 15% of total sugars and ranged from 1.22 to 4.19 g/100 g. The genotype ‘Roxana’, on the other hand, had almost identical amounts of sucrose, glucose, and fructose, with fructose content that was two to three times higher than other genotypes. The lowest sugar content was found in ‘Shakarpara’ (9.79 g/100 g), while the highest was found in ‘CITH-A-2’ (15.59 g/100 g). This is consistent with previous findings by Fan et al. [11] and Akin et al. [28]. Furthermore, the composition of individual sugars in the current study agrees with that documented by Fan et al. [11] for different Chinese apricot genotypes.

### 3.7. Sweetness Index (SI)

Individual sugars differ in sweetness, with fructose perceived as sweeter than sucrose and sucrose perceived as sweeter than glucose [15]. The sweetness is important to apricot consumers and breeders, and it also leads to market acceptance of the fruit [5]. The sweetness index (SI) ranged from 13.58 to 22.30 in the current study (Table 3). ‘CITH-A-2’ had the highest SI (22.30), followed by ‘CITH-A-1’ (22.24) and ‘Shakarpara’ (13.58). Our findings are consistent with those published SIs for Spanish apricot genotypes ranging from 8.5 to 15.9 [29]. Despite the fact that SI determines taste, the final perception of fruit sweetness is influenced by the presence of other compounds such as phenolics and other aroma compounds [11].

### 3.8. Organic Acids

**Figure 3:**
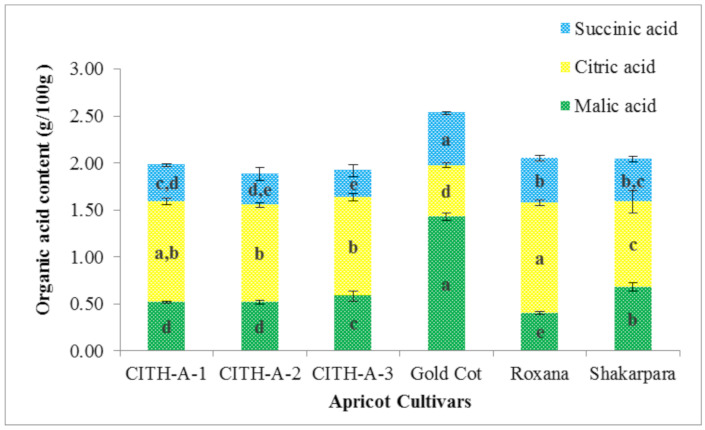
Organic acids content of apricot genotypes from India. The same letter (a, b, c, or d) inside a similarly coloured block indicates no statistically significant differences in values (*p* < 0.05).

Organic acids (OA) and sugars contribute significantly to the sensory consistency of fruits by providing a pleasant taste and aroma [20]. As shown in Figure 3, citric acid comprised approximately 55% of the OA present and ranged from 0.55 to 1.17 g/100 g, followed by malic acid, which comprised approximately 25% of the OA and ranged from 0.40 to 1.43 g/100 g, and succinic acid, which comprised approximately 20% of the OA and ranged from 0.329 to 0.56 g/100 g. The highest concentration of citric acid was found in ‘Roxana’, whereas the highest concentration of malic and succinic acids was found in ‘Gold Cot’. The malic acid content in ‘Gold Cot’ was two to three times higher than in other genotypes. Malic acid is the main contributor to fruit sourness despite being the second most abundant acid in apricots [11]. Biologically, OA play important roles. They inhibit the growth of microorganisms, which aids in the preservation of fruits. Moreover, OA have the ability to diffuse through cell membranes and dissociate to subsequent ions and protons, which lead to the acceleration of cell metabolic disorders caused by increased intercellular acidity. Furthermore, because of their ability to chelate metals, OA may serve as antioxidants and, thus, be labelled as preventive or synergistic [20]. Furthermore, these OA can aid in the stabilisation of water-soluble vitamins B and C, the enhancement of appetite and digestion, and the absorption of minerals such as potassium, copper, zinc, iron, and calcium [31].

The concentration of ascorbic acid ranged from 4.35 mg/100 g in ‘Shakarpara’ to 15.71 mg/100 g in ‘Roxana’ (Table 3). Apricot contained low amounts of ascorbic acid, which is consistent with previous reports by Fan et al. [11] and Roussos et al. [15] for apricot genotypes from China (7 to 18 mg/100 g) and Greece (11 to 47 mg/100 g), respectively.

**Figure 4:**
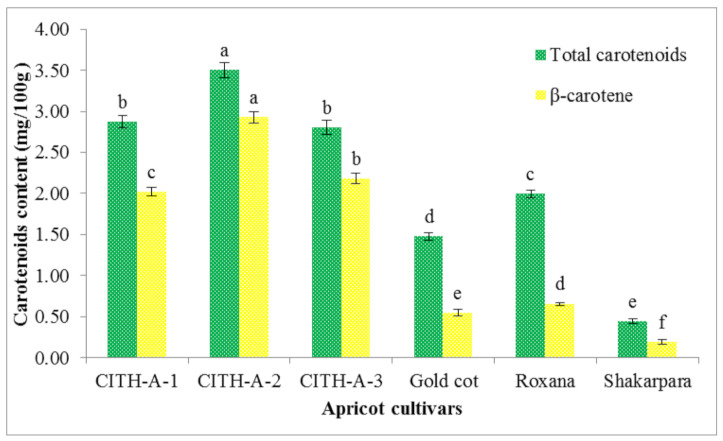
Total carotenoids and β-carotene content of apricot genotypes from India. The same letter (a, b, c, d, e or f) above a similarly coloured column indicates no significant differences in values (*p* < 0.05).

Apricots are high in carotenoids, which influence the colour and visual appearance of the fruit; the colour of the fruit can vary from yellow to orange depending on the carotenoids content [7,32]. Carotenoids are also essential dietary sources of vitamin A. In the genotypes, total carotenoids content ranged from 0.44 mg/100 g in ‘Shakarpara’ to 3.50 mg/100 g in ‘CITH-A-2’ (Figure 4). Similarly, Kafkaletou et al. [12] estimated that the total carotenoids content of Greek apricots ranged from 0.755 to 2.740 mg/100 g. Many researchers have previously identified variations in apricot total carotenoids [7,13]. Carotenoids have antioxidant properties and can protect the cell membrane from oxidative stress. Carotenoids content varies due to differences in climate, variety, geographical origin, harvest year, and cultivation methods [14].

The β-carotene content of apricot genotypes varied significantly, ranging from 0.19 to 2.93 mg/100 g (Figure 4). Among the genotypes, ‘CITH-A-2’ had the most β-carotene (2.93 mg/100 g) and ‘Shakarpara’ had the least (0.19 mg/100 g). As a result, β-carotene accounted for 33 to 84% of the total carotenoids present. The findings are consistent with previous studies that found high levels of β-carotene in apricot fruit [8,12,13,14]. Akin et al. [28] noticed that β-carotene accounted for 34 to 69% of total carotenoids in orange apricots. However, according to Kafkaletou et al. [12], β-carotene accounted for 87 to 97% of total carotenoids in apricots in Greece. β-carotene is the precursor of vitamin A and involved in retina health. It was also involved in the defence system against oxidative stress in human tissues [33].

### 3.9. Proximate Composition

The proximate composition of apricot genotypes is presented in Table 3. The genotypes had a low protein and fat content, as seen in the table. Protein content ranged from 0.24 g/100 g in ‘CITH-A-1’ to 0.61 g/100 g in ‘CITH-A-3’, while fat content varied from 0.11 g/100 g in ‘Roxana’ to 0.27 g/100 g in ‘Shakarpara’. The variation in the protein and fat content was determined to be statistically insignificant (*p* < 0.05). Our findings confirm the previous report by Fratianni et al. [32]. However, higher amounts of protein (1.12 to 1.39%) and fat (0.31 to 0.54%) were recorded by Ali et al. [14] in apricot genotypes grown in Pakistan.

Furthermore, partial differences in fibre content (1.02 to 1.51%) and ash content (0.31 to 0.40 g/100 g) were found in the genotypes studied. Recently, there has been a rise in global awareness of the importance of minerals and fibres in one’s daily diet. Minerals and fibres are considered important for sustaining wellbeing and proper physiological processes when consumed on a daily basis. The apricot genotypes in the present study provided a substantial amount of fibres and minerals. Our results are comparable to those reported by Fratianni et al. [32]. However, elsewhere higher amounts of crude fibre (1.78 to 2.57%) and ash content (1.39 to 2.44%) were recorded by Ali et al. [14]. The variation in proximate composition could be influenced by climate, variety, geographical origin, harvest year, and cultivation practices.

### 3.10. Mineral Content

The composition of mineral content of different genotypes differed significantly (*p* < 0.05), (Table 4). Potassium was the most abundant mineral, with concentrations ranging from 1430.07 to 2202.69 mg/100 g dwb, followed by phosphorous (74.91 to 249.66 mg/100 g dwb), sodium (9.32 to 13.62 mg/100 g dwb), iron (2.69 to 6.97 mg/100 g dwb), copper (0.31 to 2.73 mg/100 g dwb), zinc (0.52 to 2.28 mg/100 g dwb) and manganese which was the minor mineral (0.16 to 0.83 mg/100 g dwb). Humans require minerals to meet their physiological needs. Manganese is integrated with arginase and superoxide dismutase enzymes and also plays an important role as a co-factor of certain enzymes. Iron is a key component of haemoglobin as a core ion. Zinc is a mineral that plays a role in the body’s immune system [34]. Potassium is an electrolyte that aids in the maintenance of proper fluid balance, regulates heartbeat, maintains normal blood pressure, and lowers the risk of stroke [32]. Our findings revealed that apricot genotypes contained significant amounts of minerals; however, the genotypes ‘CITH-A-3’ and ‘CITH-A-2’ were found to be an abundant source of iron, suggesting that they could be a potential source of iron deficiency, particularly for pregnant women [35]. Previously, Ali et al. [14], Gergely et al. [34], and Akin et al. [28] found a similar pattern in apricots grown in Pakistan, Hungary, Morocco, and Turkey, respectively, with few differences in the values. The potassium content reported by Ali et al. [14] was between 2040 and 3000 mg/100 g dwb; similarly, iron content reported was 5.14–12.20 mg/100 g dwb which was lesser than reported in our study. The difference in the mineral content may be due to the difference in the location, climatic condition, soil type and genotypes used in the study.

### 3.11. Total Phenolics Content (TPC)

Phenolics and flavonoids are essential measures of nutraceutical quality and have been linked to the treatment of a variety of chronic diseases, including cancer, cardiovascular disease, and neurodegeneration. TPC content varied by more than threefold, ranging from 25.31 to 89.95 mg GAE/100 g (Table 5). Among genotypes, ‘Roxana’ had the highest TPC value (89.95 mg GAE/100 g), while ‘Shakarpara’ had the lowest (25.31 mg GAE/100 g). Wani et al. [1] and Leccese et al. [4] previously reported similar findings in apricot genotypes from India and Italy, respectively. Similarly, Kafkaletou et al. [12] and Carbone et al. [13] recorded a spectrum of TPC in various apricot genotypes ranging from 33.5 to 113.4 and 64.3 to 208.3 mg GAE/100 g, respectively. In another study, Ruiz et al. [36] reported an average of 62.1–79.2 mg GAE/100 g of phenolics in the edible portion (91% flesh and 9% peel) of white, yellow, light orange and orange coloured varieties of apricot from Spain. The authors also concluded that peels of apricot have higher content of phenolic compounds (procyanidin, hydroxycinnamic acid and flavonols) than flesh. However, a high phenolic content is associated with browning reactions caused by chemical and enzymatic reactions, resulting in a brownish colour in the manufactured product [12].

### 3.12. Total Flavonoid Content (TFC)

TFC amounts in apricot genotypes ranged from 5.00 mg CE/100 g in ‘Gold Cot’ to 15.46 mg CE/100 g in ‘CITH-A-3’ (Table 5). Our results are consistent with those reported by Carbone et al. [13], who reported TFC values ranging from 1.9 to 12.0 mg CE/100 g for different apricot genotypes. Kafkaletou et al. [12] and Wani et al. [1] found TFC values ranging from 16.87 to 41.42 and 12.2 to 36.2 mg/100 g in apricot genotypes grown in Greece and India, respectively.

### 3.13. Total Antioxidant Activity (AOX)

Antioxidant activity (AOX) was measured using various assays (namely, CUPRAC, FRAP, and DPPH) to detect a wide variety of compounds with different mechanisms in the plant matrix, including synergistic or antagonistic effects. Thus, varying the AOX assay with specific mechanisms, reaction pH, time, and temperature could provide significant benefits in terms of precision, performance, simplicity, and ease of use [37]. The CUPRAC assay yielded values ranging from 1.65 to 14.40 µmol TE/g, the DPPH assay yielded values ranging from 2.00 to 7.84 µmol TE/g, and the FRAP assay yielded values ranging from 3.61 to 12.03 µmol TE/g (Table 5). The CUPRAC values were found to be significantly higher than those of the FRAP and DPPH assays, which may be due to CUPRAC’s higher sensitivity to the presence of flavonoids, specifically quercetin and kaempferol. Flavonoids’ antioxidant potential is slightly influenced by their total OH-group material, especially the o-dihydroxy moiety in the B-ring [38]. Several researchers have previously reported similar findings [12,13]. Because of its lower redox potential, the CUPRAC assay has many benefits and is more selective.

Our findings for AOX are consistent with those of Kafkaletou et al. [12], Ali et al. [14], and Sochor et al. [6] for apricot genotypes grown in Greece, Pakistan, and the Czech Republic, respectively, with some variations in the values. Kafkaletou et al. [12], reported antioxidant parameters in the range of 57.79 to 248.40 μmol TE/100 g fresh weight. The difference in the antioxidant capacity may be due to the difference in the location, climatic condition, soil type, content of bioactive compounds, and genotypes used in the study. Overall, the genotypes ‘Roxana’ and ‘Shakarpara’ had the highest and lowest AOX levels, respectively. It is now well established that high AOX levels are primarily due to high levels of phenolic and flavonoid compounds, both of which have potential health benefits. AOX is now considered as an appropriate index for assessing the nutraceutical content of fruit.

**Figure 5:**
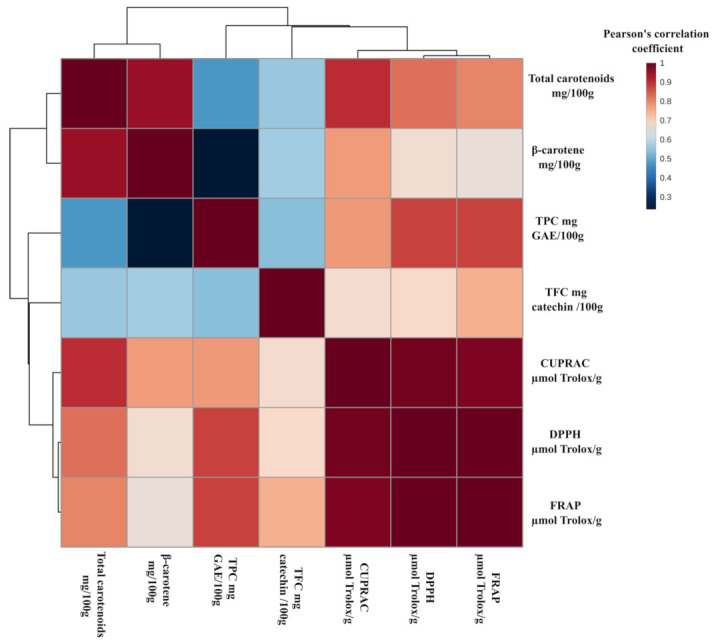
A heat map depicting Pearson’s correlation coefficients between TPC, TFC, CUPRAC, DPPH, FRAP, total carotenoids, and β-carotene. TPC—total phenolics content (mg GAE/100 g); TFC—total flavonoids content (mg CE/100 g); CUPRAC—cupric ion antioxidant reducing capacity (µmol TE/g); FRAP—ferric reducing antioxidant power (µmol TE/g); DPPH—2,2-diphenyl-1-picrylhydrazyl (µmol TE/g); GAE—gallic acid equivalents; CE—catechin equivalent; TE—Trolox equivalent.

The correlation between TPC, TFC, CUPRAC, DPPH, FRAP, total carotene, and β-carotene was further explored by measuring Pearson’s correlation coefficient between these traits (Figure 5). TPC correlated significantly with CUPRAC (r = 0.768, *p* < 0.001), DPPH (r = 0.839, *p* < 0.001), and FRAP (r = 0.863, *p* < 0.001). Several researchers obtained similar findings [39]. This strong positive correlation is due to phenolics’ ability to accept an electron, resulting in the formation of substantially stable phenoxyl radicals and, as a result, disintegrating the chain of oxidation reactions [40].

Similarly, TFC had a significant positive correlation with CUPRAC (r = 0.659, *p* < 0.001), DPPH (r = 0.666, *p* < 0.001), and FRAP (r = 0.744, *p* < 0.001). A number of researchers reported similar findings. Flavonoids exhibit antioxidant properties by inhibiting the development of reactive oxygen species (ROS) by chelation of trace elements involved in free radical production or enzyme inhibition. Flavonoids have been found to inhibit ROS-producing enzymes such as NADH oxidase, mitochondrial succinoxidase, Glutathione S-Transferase, microsomal monooxygenase, lipoxygenase, and cyclooxygenase.

Flavonoids may also chelate copper and iron, which are potential ROS enhancers [41]. Total carotene was found to be positively correlated with CUPRAC (r = 0.898, *p* < 0.001), DPPH (r = 0.798, *p* < 0.001), and FRAP (r = 0.800, *p* < 0.001). This correlation is due to the fact that carotenoids act as effective antioxidants by scavenging peroxyl radicals and singlet molecular oxygen. The singlet oxygen energy is passed to the carotenoid molecule, resulting in ground state oxygen and an excited carotene molecule. The carotenoid then returns to its ground state by dissipating more energy into the surrounding medium [42]

### 3.14. Heat Map Analysis

**Figure 6:**
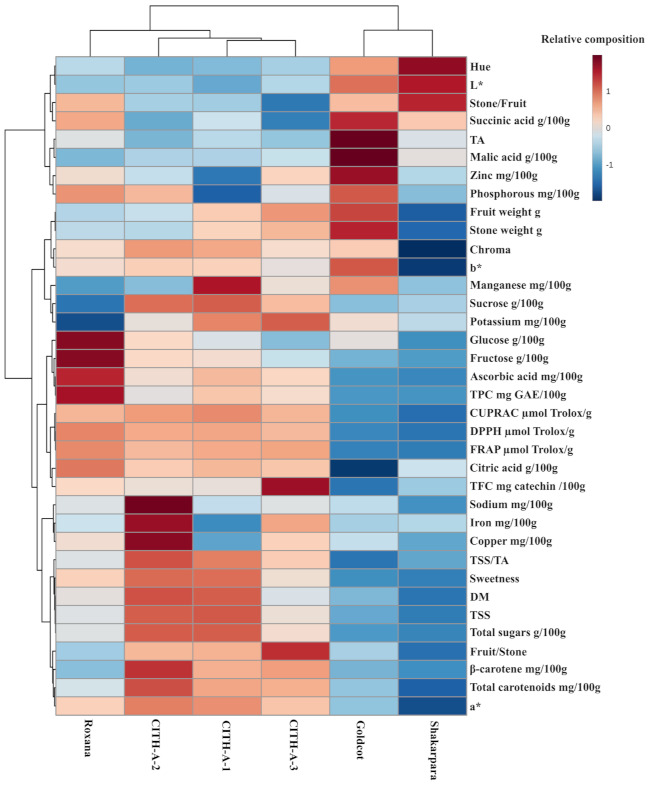
Heat map analysis of apricot genotypes and parameters. The genotypes and parameters are presented in ‘X’ and ‘Y’ dimensions, respectively. The blue–red colour map visualizes the relative value of the parameter for each genotype in a scale of low value (intensity of blue), to high value (intensity of red). L*—lightness; a*—redness; b*—yellowness; TA—titratable acidity; TFC—total flavonoids content; TPC—total phenolics content; DM—dry matter, TSS—total soluble solids; CITH—Central Institute of Temperate Horticulture; CUPRAC—cupric ion antioxidant reducing activity; FRAP—ferric reducing antioxidant power); DPPH—2,2-diphenyl-1-picrylhydrazyl.

**Figure 7:**
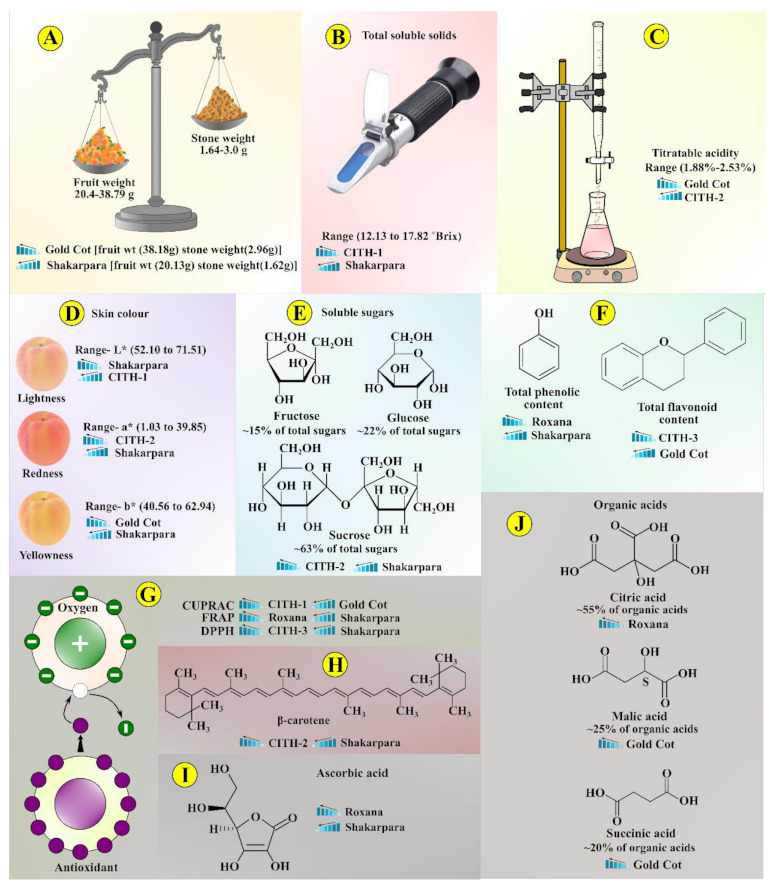
Graphical illustration indicating the evaluated attributes and their highest and lowest amount in the specific variety. (**A**) Fruit and stone weight; (**B**) total soluble solids; (**C**) titratable acidity; (**D**) skin colour where L* (lightness), a* (redness) and b* (yellowness); (**E**) soluble sugars; (**F**) phenolic and flavonoid content; (**G**) antioxidant activity through CUPRAC, FRAP and DPPH; (**H**) β-carotene content; (**I**) ascorbic acid content; (**J**) organic acid content.

The heat map (Figure 6) is an advanced method for visualising variations in samples and assisting in the drawing of fast and precise conclusions relevant to efficient data utilisation. The distinct characteristics of each genotype are depicted in an efficient, basic, and concise way. The heat map, for example, revealed that genotypes ‘CITH-A-1’ and ‘CITH-A-2’ had high levels of DM, TSS/TA, sugars, SI, minerals, carotenoids, and AOX. CITH-A-3, on the other hand, demonstrated good balance in all parameters, as well as a high flavonoids and carotenoids content. The genotype ‘Roxana’ was distinguished by high amounts of TPC, AOX, fructose, citric acid, and ascorbic acid, as well as average to high levels in the remaining parameters. The organic acid content of ‘Gold Cot’ was high, with relatively large-sized fruit and stone, but its content of other parameters was low. ‘Shakarpara’ was the genotype with the lowest overall content as compared to the other genotypes. Figure 7 illustrates the measured attributes and their highest and lowest amounts in the specific variety.

## 4. Conclusions

A comprehensive evaluation of nutritional and nutraceutical attributes of apricot genotypes commercially grown in India was attempted for the first time. Dry matter, minerals, sugars, β-carotene, phenolics, and antioxidant activity were found to vary significantly between genotypes. Genotypes exhibited a relevant source of nutraceutical compounds such as β-carotene (i.e., ‘CITH-A-2’ and ‘CITH-A-1’), phenolics (i.e., ‘Roxana’), and flavonoids (i.e., ‘CITH-A-3’). Furthermore, the mineral profile showed that apricots are a good source of potassium and iron. While the sugars and organic acids profiles revealed that sucrose and citric acid predominated in apricot genotypes. The ‘CITH-A-1’ and ‘CITH-A-2’ genotypes were associated with high DM and carotenoids and appear to be suitable for dehydrating apricots. ‘Roxana’ and ‘CITH-A-3’ offered great potential for the fresh consumer market. Whereas ‘Shakarpara’ and ‘Gold Cot’ are characterised by high acidity and moisture content, they may be more appropriate for juice processing.

## Figures and Tables

**Table 1:** Variation in physicochemical composition of apricot genotypes.

Genotypes	Fwt (g)	Swt (g)	Fwt/Swt	DM (%)	TSS	TA (%)	TSS/TA
CITH-A-1	32.40 ± 1.12 ^c^	2.42 ± 0.09 ^b^	13.23 ± 0.32 ^a,b^	18.91 ± 0.71 ^a^	17.82 ± 0.59 ^a^	1.98 ± 0.06 ^b^	9.00 ± 0.36 ^a^
CITH-A-2	28.40 ± 0.98 ^d^	2.12 ± 0.08 ^c^	13.21 ± 0.32 ^a,b^	19.11 ± 0.57 ^a^	17.77 ± 0.54 ^a^	1.88 ± 0.11 ^b^	9.47 ± 0.66 ^a^
CITH-A-3	34.93 ± 1.20 ^b^	2.53 ± 0.10 ^b^	13.65 ± 0.33 ^a^	16.07 ± 0.38 ^b^	15.37 ± 0.43 ^b^	1.92 ± 0.15 ^b^	8.04 ± 0.49 ^b^
Gold Cot	38.18 ± 1.31 ^a^	2.96 ± 0.11 ^a^	12.73 ± 0.30 ^b,c^	14.54 ± 0.51 ^c^	13.10 ± 0.46 ^c^	2.53 ± 0.07 ^a^	5.18 ± 0.19 ^e^
Roxana	27.57 ± 0.95 ^d^	2.14 ± 0.08 ^c^	12.71 ± 0.30 ^b,c^	16.27 ± 0.65 ^b^	14.99 ± 0.57 ^b^	2.05 ± 0.08 ^b^	7.31 ± 0.01 ^c^
Shakarpara	20.13 ± 0.69 ^e^	1.62 ± 0.06 ^d^	12.28 ± 0.29 ^c^	13.07 ± 0.39 ^c^	12.13 ± 0.32 ^d^	2.04 ± 0.07 ^b^	5.95 ± 0.06 ^d^
CD (0.05)	1.89	0.16	0.56	0.98	0.87	0.17	0.67

Values are a mean of three replicates ± standard deviation. All values are on fresh weight basis. Same superscript (a, b, c, d, or e) in the same column represents no significant differences between values (*p* < 0.05). CITH—Central Institute of Temperate Horticulture; Fwt—fruit weight; Swt—stone weight; DM—dry matter; TSS—total soluble solids; TA—titratable acidity.

**Table 2:** Variation in skin colour of apricot genotypes (reflectance measurements L*, a*, b*, hue angle, and chroma).

Genotypes	L*	a*	b*	H°	C*
CITH-A-1	52.10 ± 1.76 ^b^	38.18 ± 0.89 ^a^	56.58 ± 1.04 ^b^	55.99 ± 0.51 ^c,d^	68.26 ± 1.22 ^a,b^
CITH-A-2	54.65 ± 4.81 ^b^	39.85 ± 2.82 ^a^	56.67 ± 4.69 ^b^	54.84 ± 5.33 ^d^	69.48 ± 2.08 ^a^
CITH-A-3	55.94 ± 4.17 ^b^	32.56 ± 2.44 ^b^	54.42 ± 0.47 ^b^	59.13 ± 2.08 ^c,d^	63.45 ± 0.92 ^c^
Gold Cot	67.19 ± 0.73 ^a^	16.82 ± 2.45 ^c^	62.94 ± 0.88 ^a^	75.06 ± 1.89 ^b^	65.17 ± 1.45 ^b,c^
Roxana	54.22 ± 0.92 ^b^	30.89 ± 2.82 ^b^	55.40 ± 0.49 ^b^	60.90 ± 1.99 ^c^	63.46 ± 1.81 ^c^
Shakarpara	71.51 ± 3.6 ^a^	1.03 ± 1.19 ^d^	40.56 ± 3.74 ^c^	88.43 ± 1.88 ^a^	40.59 ± 3.70 ^d^
CD (0.05)	5.24	4.89	4.50	4.82	3.69

Values are a mean of three replicates ± standard deviation. Same superscript (a, b, c, or d) in the same column represents no significant differences between values (*p* < 0.05). CITH—Central Institute of Temperate Horticulture; H°—hue angle; C*—chroma; L* (lightness); a* (redness); b* (yellowness).

**Table 3:** Variation in the approximate composition and sweetness of apricot genotypes.

Genotype	Protein g/100 g	Fat g/100 g	Fibre g/100 g	Ash g/100 g	Total Sugars g/100 g	SI	Ascorbic Acid (mg/100 g)
CITH-A-1	0.61 ± 0.16	0.24 ± 0.14	1.23 ± 0.37	0.37 ± 0.16	15.54 ± 0.57 ^a^	22.24 ± 0.87 ^a^	11.52 ± 0.34 ^b^
CITH-A-2	0.55 ± 0.24	0.18 ± 0.11	1.51 ± 0.54	0.37 ± 0.09	15.59 ± 0.57 ^a^	22.30 ± 0.71 ^a^	9.90 ± 0.26 ^c^
CITH-A-3	0.24 ± 0.20	0.19 ± 0.09	1.41 ± 0.44	0.40 ± 0.08	13.16 ± 0.32 ^b^	18.77 ± 0.44 ^b^	10.50 ± 0.92 ^c^
Gold Cot	0.43 ± 0.22	0.24 ± 0.16	1.02 ± 0.35	0.38 ± 0.12	10.27 ± 0.44 ^c^	14.15 ± 0.43 ^c^	4.83 ± 0.17 ^d^
Roxana	0.54 ± 0.15	0.11 ± 0.12	1.27 ± 0.53	0.31 ± 0.05	12.65 ± 0.49 ^b^	19.54 ± 0.80 ^b^	15.71 ± 0.39 ^a^
Shakarpara	0.40 ±0.28	0.27 ± 0.14	1.43 ± 0.50	0.34 ± 0.06	9.79 ± 0.25 ^c^	13.58 ± 0.35 ^c^	4.35 ± 0.24 ^d^
CD (0.05)	NS	NS	NS	NS	0.81	1.13	0.82

Total sugars represent the sum of individual sugars, i.e., glucose, fructose and sucrose. Values are a mean of three replicates ± standard deviation. Same superscript (a, b, or c) in the same column represents no significant differences between values (*p* < 0.05). All values are on a fresh weight basis. NS—indicates non-significant differences between values (*p* < 0.05); CITH—Central Institute of Temperate Horticulture; SI—sweetness index.

**Table 4:** Variation in mineral content of different apricot genotypes.

Genotypes	Potassium (K)	Phosphorus (P)	Sodium (Na)	Iron (Fe)	Copper (Cu)	Manganese (Mn)	Zinc (Zn)
CITH-A-1	1787.13 ± 22.85 ^b^	74.91 ± 5.85 ^d^	9.32 ± 0.03 ^c^	2.69 ± 0.12 ^d^	0.31 ± 0.01 ^f^	0.83 ± 0.06 ^a^	0.52 ± 0.01 ^d^
CITH-A-2	1586.00 ± 73.25 ^c^	162.56 ± 5.28 ^c^	13.62 ± 0.67 ^a^	6.97 ± 0.61 ^a^	2.73 ± 0.08 ^a^	0.22 ± 0.06 ^d^	0.94 ± 0.06 ^c^
CITH-A-3	2157.79 ± 50.30 ^a^	162.15 ± 2.50 ^c^	11.56 ± 0.20 ^b^	6.39 ± 0.03 ^b^	1.67 ± 0.03 ^b^	0.50 ± 0.01 ^b^	1.38 ± 0.14 ^b^
Gold Cot	2105.95 ± 75.21 ^a^	249.66 ± 4.25 ^a^	12.11 ± 1.23 ^b^	4.75 ± 0.06 ^c^	1.16 ± 0.07 ^d^	0.80 ± 0.01 ^a^	2.28 ± 0.10 ^a^
Roxana	1430.07 ± 54.26 ^d^	204.81 ± 8.40 ^b^	11.32 ± 0.61 ^b^	4.74 ± 0.14 ^c^	1.46 ± 0.06 ^c^	0.16 ± 0.01 ^d^	1.30 ± 0.06 ^b^
Shakarpara	2202.69 ± 39.65 ^a^	162.54 ± 3.08 ^c^	11.24 ± 0.35 ^b^	5.51 ± 0.16 ^c^	0.47 ± 0.03 ^e^	0.33 ± 0.04 ^c^	1.31 ± 0.10 ^b^
CD (0.05)	99.03	9.37	1.14	0.48	0.09	0.06	0.15
RDA for adults	3500	1200	2400	15	1.5 to 3	2 to 5	15

Values are a mean of three replicates ± standard deviation. Results were expressed as mg per 100 g on a dry weight basis. The same superscript (a, b, c, d, or e) in the same column represents no significant differences between values (*p* < 0.05). CITH—Central Institute of Temperate Horticulture; RDA for adults—recommended dietary allowance for adults and pregnant women mg/day as given by the National Academy of Science [35].

**Table 5:** Variation in total phenolics, flavonoid, and antioxidant activity in different apricot genotypes.

Genotypes	Total Phenolics (mg GAE/100 g)	Total Flavonoids (mg CE/100 g)	Antioxidants Activity (µmol TE/g)		
			CUPRAC	DPPH	FRAP
CITH-A-1	60.42 ± 2.43 ^b^	9.81 ± 0.69 ^b^	14.40 ± 0.73 ^a^	7.25 ± 1.08 ^a^	11.13 ± 0.71 ^a,b^
CITH-A-2	50.30 ± 1.74 ^c^	9.90 ± 0.95 ^b^	13.80 ± 0.72 ^a^	7.15 ± 0.67 ^a^	10.65 ± 0.40 ^b^
CITH-A-3	55.35 ± 6.48 ^b,c^	15.46 ± 0.63 ^a^	12.74 ± 0.64 ^b^	6.84 ± 0.92 ^a^	11.32 ± 0.67 ^a,b^
Gold Cot	26.24 ± 1.36 ^d^	5.00 ± 0.83 ^d^	3.45 ± 0.39 ^c^	2.47 ± 0.24 ^b^	3.88 ± 0.21 ^c^
Roxana	89.95 ± 2.39 ^a^	10.46 ± 0.43 ^b^	12.73 ± 0.41 ^b^	7.84 ± 1.03 ^a^	12.03 ± 0.66 ^a^
Shakarpara	25.31 ± 1.82 ^d^	7.71 ± 0.64 ^c^	1.65 ± 0.26 ^d^	2.00 ± 0.23 ^b^	3.61 ± 0.22 ^c^
CD (0.05)	5.71	1.27	0.99	1.38	0.93

Values are a mean of three replicates ± standard deviation. Results were expressed on a fresh weight basis. The same superscript (a, b, c, or d) in the same column represents no significant differences between values (*p* < 0.05). CITH—Central Institute of Temperate Horticulture. CUPRAC—cupric ion antioxidant reducing activity; FRAP—ferric reducing antioxidant power; DPPH—2, 2-diphenyl-1-picrylhydrazyl; GAE—gallic acid equivalents; CE—catechin equivalent; TE—Trolox equivalent.

## Data Availability

Data available on request.
